# Supplementary material for: Transcriptome-wide identification of transient RNA G-quadruplexes in human cells
Source: Nat Commun. 2018 Nov 9;9:4730. doi: 10.1038/s41467-018-07224-8 (PMC6226477; doi:10.1038/s41467-018-07224-8)
Supplement: Supplementary file 1 — Supplementary Information [file 41467_2018_7224_MOESM1_ESM.pdf]

Transcriptome-wide identification of transient RNA G-quadruplexes in human cells

Sunny Y. Yang,[1] Pauline Lejault,[2] Sandy Chevrier,[3] Romain Boidot,[3]
A. Gordon Robertson,[4] Judy M. Y. Wong,[1,*] and David Monchaud[2,*]

[1]Faculty of Pharmaceutical Sciences, The University of British Columbia, Vancouver, Canada.
[2]Institut de Chimie Moléculaire, ICMUB CNRS UMR6302, UBFC Dijon, France.
[3]Platform of Transfer in Cancer Biology, Centre Georges-François Leclerc, Dijon, France
[4]Genome Sciences Center, BC Cancer Agency, Vancouver, Canada

SUPPLEMENTARY SECTION

-- I. Chemistry.

Material and methods. All chemicals were purchased from Sigma-Aldrich, except for Boc-^{PNA}G(Z)-OH which was purchased from ASM Research Chemicals and used without further purification. NMR spectra were recorded with a Bruker 300 Avance III NanoBay spectrometer (300 MHz for ^{1}H and 75 MHz for ^{13}C). Chemical shifts are reported in d ppm, using [D] solvents as standards. The following abbreviations are used: s: singlet, d: doublet, t: triplet, q: quartet, m: multiplet, br: broad. MALDI-TOF (Matrix-Assisted Laser Desorption/Ionization - Time of Flight) mass spectrometry was carried out using a Bruker Ultraflex II LRF 2000 spectrometer and ESI (ElectroSpray Ionization) mass spectrometry was carried out using a Bruker Daltonics AmaZon SL spectrometer. Purification: Flash chromatography was performed using the Automatic Reveleris® Flash Chromatography System (GRACE) equipped with multiple channel detection (UV (201 and/or 214 nm) and ELSD (Evaporative Light Scattering Detection)). A Reveleris® C18 RP 80 g cartridge was used, with CH_3CN/0.1 % TFA and H_2O/0.1 % TFA as eluents (linear gradient at 0% CH_3CN/0.1 % TFA (12 min), linear gradient from 0% to 10% CH_3CN/0.1 % TFA (10 min), linear gradient from 10% to 40% CH_3CN/0.1 % TFA (5 min), linear gradient from 40% to 55% CH_3CN/0.1 % TFA (5 min)) at a flow rate of 60 mL/min. Semi-preparative RP-HPLC was performed using a Thermo Beta Basic C18 column, 5 µm, 30 x 150 mm with CH_3CN/0.1 % HCO_2H and H_2O/0.1 % HCO_2H as eluents (linear gradient from 5 to 50% CH_3CN/0.1 % HCO_2H (45 min)) at a flow rate of 15 mL/min with a UV-Vis detection with an Ultimate 3000 diode array detector at 201, 214, 222, 260 nm.

The design of BioTASQ (Figure 1A, left) is based on two previously reported TASQs, *i.e.*, ^{PNA}DOTASQ (Figure 1A, center) (1) which is considered here as the parent compound, and of Pyro-DOTASQ (Figure 1A, right) (2), in which a pyrene moiety was grafted on the DOTA template. Its synthesis (Figure 1B) is performed as follows: the Pyro-DOTASQ key intermediate aminomethylcyclen 1, obtained after a described 5-step protocol from the commercially available triethylenetetramine (TETA), is coupled with biotin after activation with 2-succinimido-1,1,3,3-tetramethyluronium tetrafluoroborate (TSTU), to provide the biotin-cyclen conjugate 2; the TASQ core is subsequently constructed according to the ^{PNA}DOTASQ strategy, involving introduction of four ethyl bromoacetate arms (intermediate 3) subsequently elongated upon reaction with ethylene diamine to provide the intermediate 4 that finally reacts with *N*-Boc-protected ^{PNA}G monomers (Boc-^{PNA}G-OH) to lead, after a final acidic deprotonation step, to BioTASQ. Of note, each compound is purified by semi-prep HPLC, which proves difficult for the final compound BioTASQ, thus explaining the very low chemical yield (5%) for the very final steps.

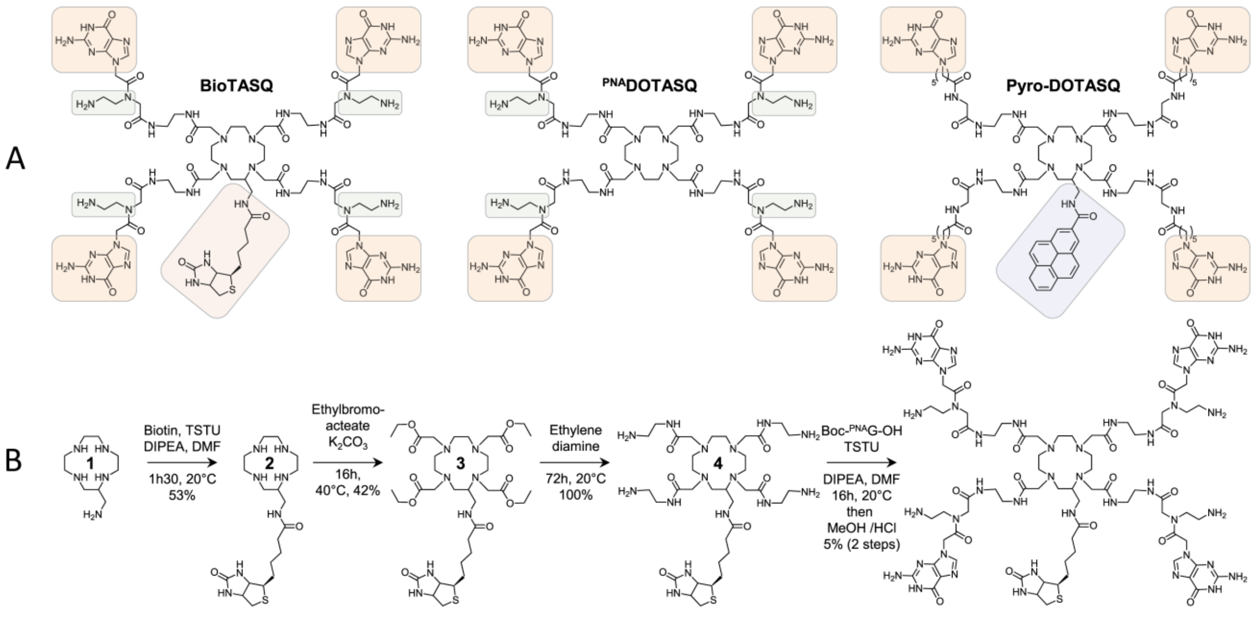

Figure 1. A. Chemical structures of BioTASQ, ^{PNA}DOTASQ and Pyro-DOTASQ. **B.** Chemical synthesis of BioTASQ.

Step 1: TSTU (1.61 g, 5.36 mmol), biotin (1.19 g, 4.87 mmol) and DIPEA (0.93 mL, 5.36 mmol) were dissolved in DMF (50 mL). The mixture was stirred at room temperature for 30 min.

A solution of **1** (cf. Note 1, 0.98 g, 4.87 mmol) and DIPEA (1.695 mL, 9.74 mmol) in DMF (25 mL) was slowly added and the mixture was stirred at room temperature for 1 hr. The solvent was evaporated under reduced pressure and the crude mixture was purified by flash chromatography to afford **2** as a white solid (1.11 g, 53%). ^1H NMR (500 MHz, d6-DMSO): δ 7.50-7.37 (dd, 1H); 4.47-4.25 (m, 2H); 3.32-2.65 (m, 24H), 2.20 (m, 2H); 1.67-1.25 (m, 8H). HR-MS (m/z): $[M+H]^+$ calcd. for $C_{19}H_{38}N_7O_2S$, 428.27; found, 428.27 (and 450.26 $[M+Na]^+$).

Step 2: To a solution of **2** (200 mg, 0.31 mmol) and K_2CO_3 (337 mg, 2.44 mmol) in acetonitrile (3 mL) was added ethylbromoacetate (138 μL, 1.25 mmol). The mixture was stirred overnight at 40°C. It was filtered through dicalite and washed with acetonitrile. The filtrate was evaporated under reduced pressure. The crude mixture was purified by semi-preparative HPLC to afford **3** as a white solid (100 mg, 42 %). ^1H NMR (500 MHz, d6-DMSO): δ 7.30 (s broad, 1H); 5.50-5.09 (m, 2H); 4.26-4.07 (m, 8H); 3.46-2.04 (m, 27H), 1.65-1.42 (m, 2H); 1.29-1.18 (m, 18H); 0.88 (m, 3H). HR-MS (m/z): $[M+H]^+$ calcd. for $C_{35}H_{62}N_7O_{10}S$, 772.42; found, 772.43 (and 794.42 $[M+Na]^+$).

Steps 3 and 4: A solution of **3** (30 mg, 0.039 mmol) in ethylene diamine (1 mL) was stirred at room temperature for 3 days. Ethylene diamine was evaporated under reduced pressure. The mixture was triturated successively in acetonitrile and diethyl ether to afford **4** as a white solid, which is directly engaged in the following step: Boc-^{PNA}G-OH (cf. note 2, 238 mg, 0.58 mmol), TSTU (175 mg, 0.58 mmol) and DIPEA (101 μL, 0.58 mmol) were dissolved in DMF (1.6 mL). The mixture was stirred at room temperature for 30 min. A solution of **4** (96 mg, 0.12 mmol) and DIPEA (40 μL, 0.23 mmol) in DMF (2 mL) was then added dropwise and the mixture was stirred overnight at room temperature. The solvent was removed under reduced pressure. The crude mixture was purified by semi-preparative HPLC to afford **5** as a white solid. ^1H NMR (500 MHz, d6-DMSO): δ 10.77 (s broad, 2H); 7.9-7.42 (m, 14H); 7.05 (s broad, 2H); 6.90-6.62 (m, 5H); 6.17 (dd, 5H); 5.32-4.84 (m, 9H); 4.26-4.03 (m, 10H); 3.52-3.07 (m, 43H), 1.82 (m, 5H); 1.39-0.99 (m, 58H). MS (MALDI-TOF, m/z): $[M+H]^+$ calcd for 2393.17, found 2393.22.

Step 5: 5 mL of a solution of methanol saturated with HCl were added to **5**. The mixture was stirred for 30 min at room temperature. Diethyl ether was added gently to precipitate the compound. The supernatant was removed and the residue was dried under reduced pressure to afford BioTASQ as a white powder (11 mg, 4.6%). ^1H NMR (500 MHz, d6-DMSO): δ 11.49 (s broad,

4H); 8.78-8.42 (m, 5H); 8.42-8.04 (2 s broad, 9H); 7.12 (s broad, 8H); 5.31-5.04 (m, 8H); 4.26-2.92 (m, water signals precluded proper interpretation, expected: 85H, 18H detectable), 1.30-1.12 (m, 2H). MS (MALDI-TOF, m/z): $[M+H]^+$ calcd for $C_{79}H_{122}N_{43}O_{18}S$, 1991.96; found 1993.09. HR-MS (m/z): $[M+2H+H2O]^{2+}$, cacld. for 1004.98451; found 1004.98318 (Δ =-1.323 ppm).

Note 1: Synthesis of aminomethylcyclen **1** (3). **Step 1**: A solution of 2,3-butanedione (88.3 g, 1.03 mol) was added dropwise to a solution of triethylenetetraamine (TETA, 150 g, 1.03 mol) and calcium hydroxide (151.8 g, 2.05 mol) in acetonitrile (6 L) at 0°C. The mixture was stirred at this temperature for 2hrs. It was then filtered over dicalite and washed with acetonitrile to afford a yellow solution. This solution was cooled down to 0°C and K_2CO_3 (283.2 g, 2.05 mol) and benzotriazole (122.1 g, 1.03 mol) were added. A solution of chloroacetaldehyde in acetonitrile (1 L) was slowly added at 0°C and the resulting mixture was stirred for 2 h. NaCN (50.3 g, 1.03 mol) was added and the mixture was stirred overnight at room temperature. The solution was filtered through dicalite and washed with acetonitrile. The filtrate was evaporated. **Step 2**: A solution of the previously prepared compound (126.9 g, 0.51 mol) in dry THF (500 mL) was slowly added to a suspension of $LiAlH_4$ (23.4 g, 0.62 mol) in dry THF (1 L) under nitrogen at –78°C. The resulting mixture was stirred overnight. Water (250 mL) was carefully added to the mixture at –78°C to neutralize the excess $LiAlH_4$. The mixture was evaporated and the residue was taken up in chloroform (1.5 L) and stirred overnight at room temperature. The mixture was filtered through dicalite and washed with chloroform. The filtrate was concentrated under reduced pressure. **Step 3**: A solution of the previously prepared compound (77.3 g, 0.30 mol) in 37% hydrochloric acid (250 mL, 3 mol, 10 equiv.) was stirred overnight at 80°C. The mixture was evaporated and triturated in Et_2O. It was filtered and washed successively with 37% HCl and diethyl ether to afford aminomethylcyclen **1** as a white powder (21.73 g, 19%). ^1H NMR (300 MHz, $CDCl_3$, 300K): δ (ppm): 2.01 (br. s., 6H), 2.40-2.80 (m, 17H).

Note 2: Synthesis of Boc-^{PNA}G-OH (1): To a solution of Boc-^{PNA}G(Z)-OH (1 g, 1.84 mmol) in methanol (50 mL) was added Pd/C (39.1 mg, 20 mol%). The suspension was stirred overnight at room temperature under H_2. The solid was then filtered over dicalite and washed with methanol.

The residue was concentrated under reduced pressure to afford Boc-^{PNA}G-OH as a white solid (540 mg, 72%). ^{1}H NMR (300 MHz, d6-DMSO, 300K): δ (ppm): 1.37 (s, 9H), 3.20-3.80 (m, 4H), 3.99 (s, 2H), 4.85 (s, 2H), 6.46 (s, 2H), 6.95 (m,1H), 7.51 (s, 1H), 10.59 (s, 1H).

-- II. FRET-melting experiments.

The nucleic acid binding capabilities of BioTASQ were investigated with the fluorescence resonance energy transfer (FRET)-melting assay (4): experiments were implemented with increasing concentrations of BioTASQ (1-10 μM) against a panel of biologically relevant doubly labeled quadruplex-forming sequences (Figure 2), namely F21T, F-myc-T and F-kit-T, which are DNA sequences found in the human telomeres and in the promoter regions of myc and kit genes, respectively, F-TERRA-T, which is a RNA sequence found in the human telomeric transcripts, along with F-DS-T, a duplex-DNA control. As seen in Figures S2, BioTASQ stabilizes the quadruplexes in a dose-response manner, with $\Delta T_{1/2}$ values up to 19.6°C (for DNA) and 12.1°C (for RNA) at 10 μM concentration, albeit modestly since very low stabilizations are obtained at 1 μM concentration ($\Delta T_{1/2}$ = 0.1-0.8°C) that is considered as the standard ligand concentration in the FRET-melting assay. Of note, in these conditions (i.e., 1 μM ligand), the parent compound ^{PNA}DOTASQ stabilizes quadruplexes more efficiently with $\Delta T_{1/2}$ values between 12.5-21.1°C; these results indicate that the introduction of the biotin tag reduced ligand affinity. We next investigated the quadruplex selectivity of BioTASQ, which is concluded to be similar to that of the parent compound ^{PNA}DOTASQ, as shown by a low F-DS-T stabilization ($\Delta T_{1/2}$ up to 1.5°C, at 10 μM concentration) and further confirmed *via* competitive FRET-melting experiments carried out with labeled quadruplexes in the presence of 15 and 50 mol. equiv. of unlabeled 26-bp duplex-DNA ds26. As seen in Figure 2, the stabilization is maintained at 78-100% and 71-87% in the presence of 15 and 50 mol. equiv. ds26, respectively, with overall better affinity for quadruplex-RNA *vs*. quadruplex-DNA. Collectively, these two series of data highlighted the fair G4 affinity but high selectivity of BioTASQ for quadruplexes (vs duplex-DNA).

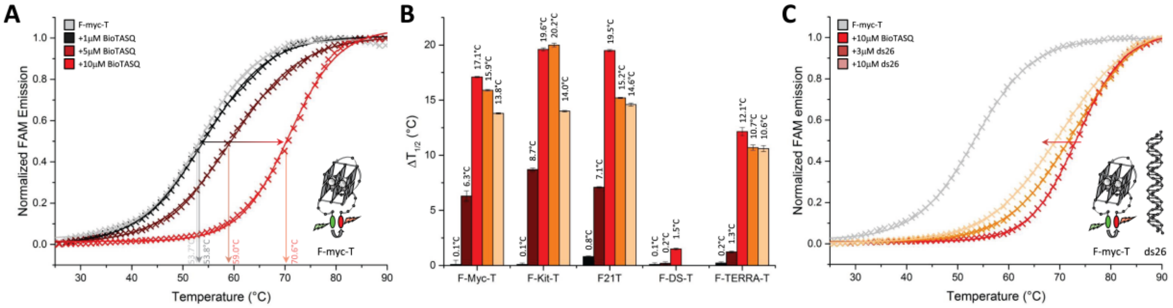

Figure 2. A. Dose-response FRET-melting curves obtained with F-myc-T (0.2mM) and increasing amounts of BioTASQ (1-10mM). **B.** Summary of $DT_{1/2}$ values with F-myc-T, F-kit-T, F21T, F-DS-T and F-TERRA-T (0.2mM) and increasing amounts of BioTASQ (1-10mM), without or with the duplex ds26 as competitor (3-10mM). **C**. Competitive FRET-melting curves obtained with F-myc-T (0.2mM), BioTASQ (10mM) and increasing amounts of ds26 (3-10mM).

The sequences of the oligonucleotides used herein are: F-21-T: FAM-d[$^{5'}G_3T_2AG_3T_2AG_3T_2AG_3^{3'}$]-TAMRA; F-myc-T: FAM-d[$^{5'}GAG_3TG_4AG_3TG_4A_2G^{3'}$]-TAMRA; F-kit-T: FAM-d[$^{5'}CG_3CG_3CGCGAG_3AG_4^{3'}$]-TAMRA); F-DS-T: FAM-d[$^{5'}TATAGCTATAT_6TATAGCTATA^{3'}$]-TAMRA; F-TERRA-T: FAM-5[$^{5'}$ $G_3U_2AG_3U_2AG_3U_2AG_3^{3'}$]-TAMRA; and the self-complementary ds26: d[$^{5'}CA_2TCG_2ATCGA_2T_2CGATC_2GAT_2G^{3'}$]. Lyophilized DNA strands (purchased from Eurogentec, Seraing, Belgium) were first diluted to 500µM in deionized water (18.2 MΩ.cm resistivity). All DNA structures were prepared in Caco.K buffer, comprised of 10 mM lithium cacodylate buffer (pH 7.2) plus 10 mM KCl/90 mM LiCl (F21T, F-DS-T) or plus 1 mM KCl/99 mM LiCl (F-MYC-T, F-KIT-T, F-TERRA-T). Quadruplex structures were prepared by mixing 40 µL of the constitutive strand (500 µM) with 8µL of a lithium cacodylate buffer solution (100 mM, pH 7.2), plus 8µL of a KCl/LiCl solution (100 mM/900 mM) and 24µL of water. The duplex structure (ds26) was prepared by mixing 40 µL of each constitutive strand (500 µM) with 16 µL of a lithium cacodylate buffer solution (100 mM, pH 7.2), plus 16µL of a KCl/LiCl solution (100 mM/900 mM) and 48 µL of water. The final concentrations were theoretically 250 and 125 µM, for mono- and bimolecular DNA structures, respectively. Actual concentration of each DNA was determined through dilution to 1 µM theoretical concentration and confirmed with UV spectral analysis at 260 nm (after 5 min at 90 °C) with the following molar extinction coefficient values: 268300 (F21T), 232000 (F-myc-T), 205600 (F-kit-T), 258900 (F-DS-T), 276700 (F-TERRA-T) and 506400 $M^{-1}.cm^{-1}$ (ds26). Higher-order

DNA structures were folded according to two procedures: (a) for the monomolecular architectures, solutions were heated (90°C, 5 min), cooled on ice (7 h) and then stored at least overnight (4°C); (b) for the folding of all other structures, the solutions were heated (90°C, 5 min), gradually cooled (65, 60, 55, 50, 40 and 30°C (30 min/step), 25°C (2 h)) and then stored at least overnight (4°C).

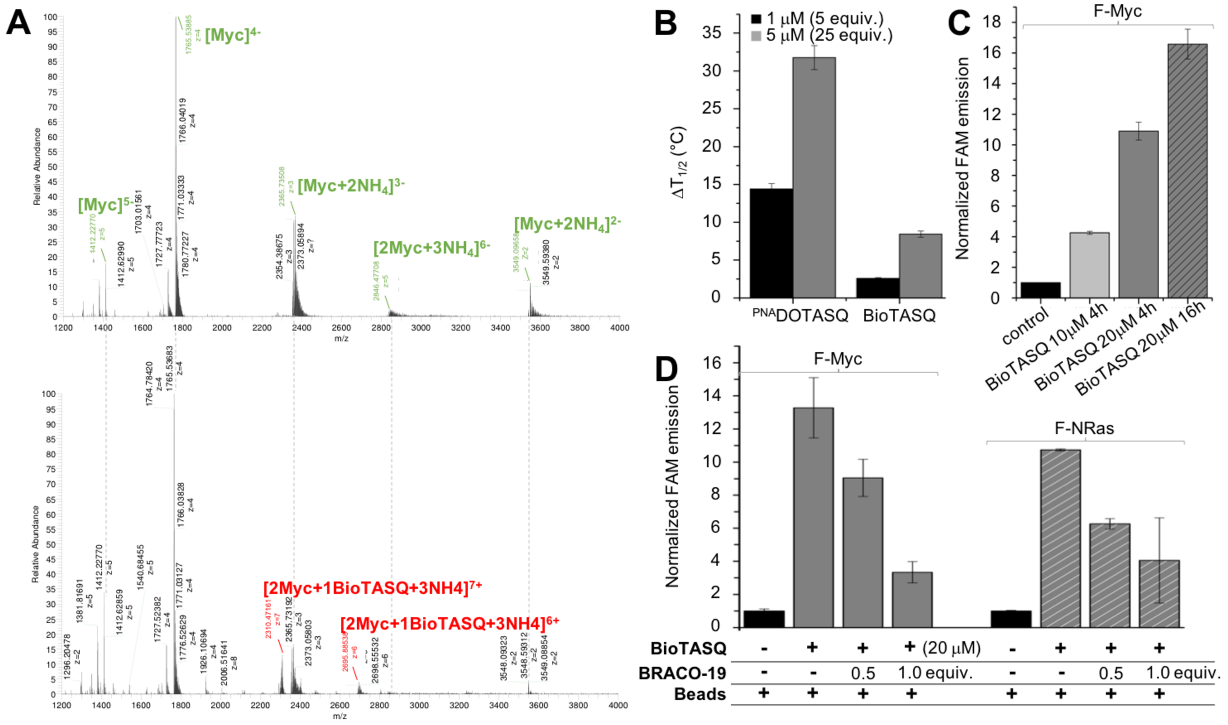

Figure 3. A) Electrospray ionization mass spectra (ESI-MS) analysis demonstrated the low affinity and stoichiometry (1:1) of association between Myc quadruplex and BioTASQ. B) Confirmation of the lower quadruplex-affinity of BioTASQ as compared to the parent molecule ^{PNA}DOTASQ. FRET-melting experiments were performed with F-Myc-T (0.2 μM) with increasing amounts of ligands (1 and 5 μM, *i.e.*, 5 and 25 mol. equiv.). C) Pull-down experiments were performed with F-Myc (1 μM) and increasing BioTASQ concentrations (10 and 20 μM) and incubation times (4 and 16h). Experiments were done with triplicates D) Competition BioTASQ (20 μM) pull-down experiments were performed with F-Myc and F-NRas (1 μM), and increasing amounts of BRACO-19 (0.5 and 1 μM). Experiments were done with triplicates. Error bars represented SD.

FRET-melting experiments were performed in 96-well format using a Mx3005P qPCR machine (Agilent) equipped with FAM filters (λ_{ex} = 492 nm; λ_{em} = 516 nm) in 100 µL (final volume) 10 mM lithium cacodylate buffer (pH 7.2) plus 10 mM KCl/90 mM LiCl (F21T, F-DS-T) or plus 1 mM KCl/99 mM LiCl (F-MYC-T, F-KIT-T, F-TERRA-T) with 0.2 µM of labeled oligonucleotide and 0,

1, 5 or 10 μM of BioTASQ. Competitive experiments were carried out with labeled oligonucleotide (0.2 μM), 10 μM BioTASQ and increasing amounts (0, 15 and 50 mol. equiv.) of the unlabeled competitor ds26. After a first equilibration step (25 °C, 30 s), a stepwise increase of 1 °C every 30 s for 65 cycles to reach 90 °C was performed, and measurements were made after each cycle. Final data were analyzed with Excel (Microsoft Corp.) and OriginPro®9.1 (OriginLab Corp.). The emission of FAM was normalized (0 to 1), and $T_{1/2}$ was defined as the temperature for which the normalized emission is 0.5; $\Delta T_{1/2}$ values are means of 2-4 experiments.

Electrospray mass spectrometry experiments were performed on a LTQ Orbitrap XL (Thermo Scientific) spectrometer equipped with Ion Max source and HESI-II probe in the negative ion mode, according to the previously described protocol (5). Myc (d[$^{5'}GAG_3TG_4AG_3TG_4A_2G^{3'}$], 10 μM) and the corresponding Myc:BioTASQ mixture (1:1 ratio) were prepared in 100 mM ammonium acetate buffer and equilibrated at 25°C for 1 hour. To obtain a stable electrospray signal, 20% of methanol were added to the solution just before injection. The solutions were injected with syringe pump at a flow rate of 5 μL/min. The full scan mass was recorded in 600-4000 m/z range. The following tuning parameters were used: heater temperature = 50°C, spray voltage = 4.0 kV, capillary temperature = 275°C, Tube lens = -160.00 (negative ion mode) and the capillary voltage varied between -35.00 V and -60.00V.

-- III. Dose-response Profiling of G4 ligands.

MCF7 cells were cultured with 5%FBS in DMEM (Hyclone), washed with PBS (Gibco) and trypsinized with 0.025% Trypsin/EDTA (Gibco). MCF7 cells were seeded at 3000/well in a 96-well flat bottom plate (Corning). The cells were treated with serial dilutions of indicated G4 ligands. Growth kinetics and cell morphology were recorded using the Essenbio IncuCyte ZOOM live-cell monitoring system (6). Dose response curves were generated using Graphpad Prism. The inhibitory dose 50 (or lethal dose 50, or LD_{50}) at which 50% growth inhibition occurred was calculated from the generated dose response curves. Doses between IC_{15} (or LD_{15}) to IC25 (or LD_{25}) were used for subsequent experiments.

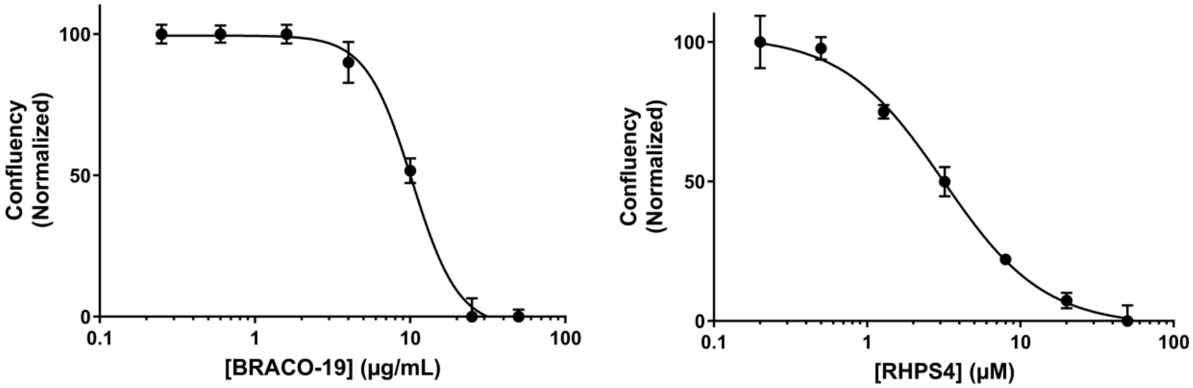

MCF7	
Treatment	BRACO-19
IC50	10.3 µg/ml
Hill Slope	2.93
IC25	6.7 µg/ml
IC15	5.1 µg/ml

MCF7	
Treatment	RHPS4
IC50	3.13 µM
Hill Slope	1.32
IC25	1.5 µM
IC15	1.1 µM

Figure 4. Dose response profiles for BRACO-19 and RHPS4 in MCF7 cells generated from IncuCyte ZOOM live-cell monitoring system. Experiments were conducted with three biological replicates. The IC50, hill slope, IC25 and IC15 are listed in the tables below. Error bars represent SEM.

-- IV. Additional G4RP & G4RP-seq results.

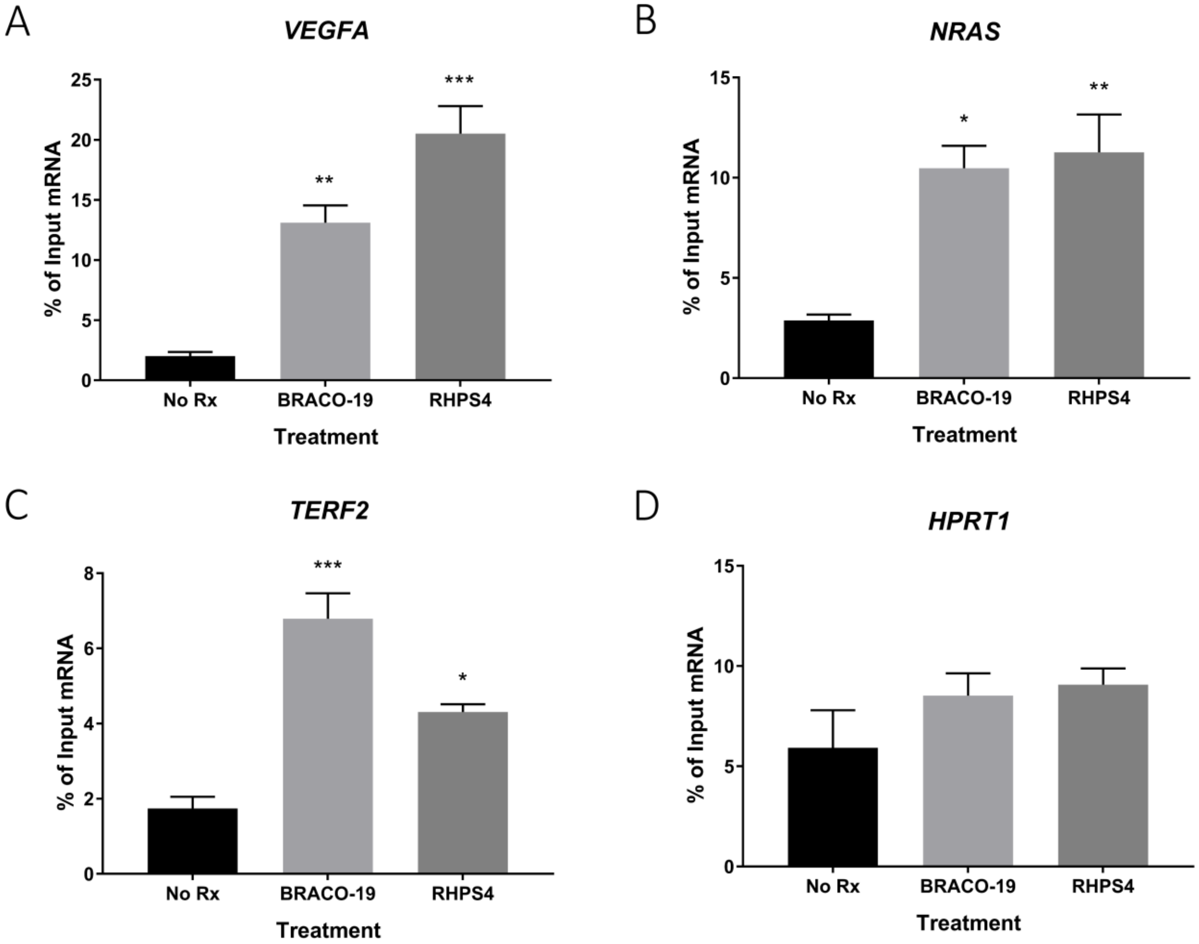

Figure 5. BioTASQ G4RP signals were quantified with RT-qPCR measurements of (A) VEGFA, (B) NRAS, (C) TERF2 or (D) HPRT1 mRNA levels in untreated (No Rx), BRACO-19-treated or RHPS4-treated MCF7 cells. Values are normalized to their individual input control used for the BioTASQ pull down. One-way ANOVA was performed against No Rx control. *$p<0.05$, **$p<0.01$, ***$p<0.001$. Experiments were conducted with three biological replicates. Error bars represent SEM.

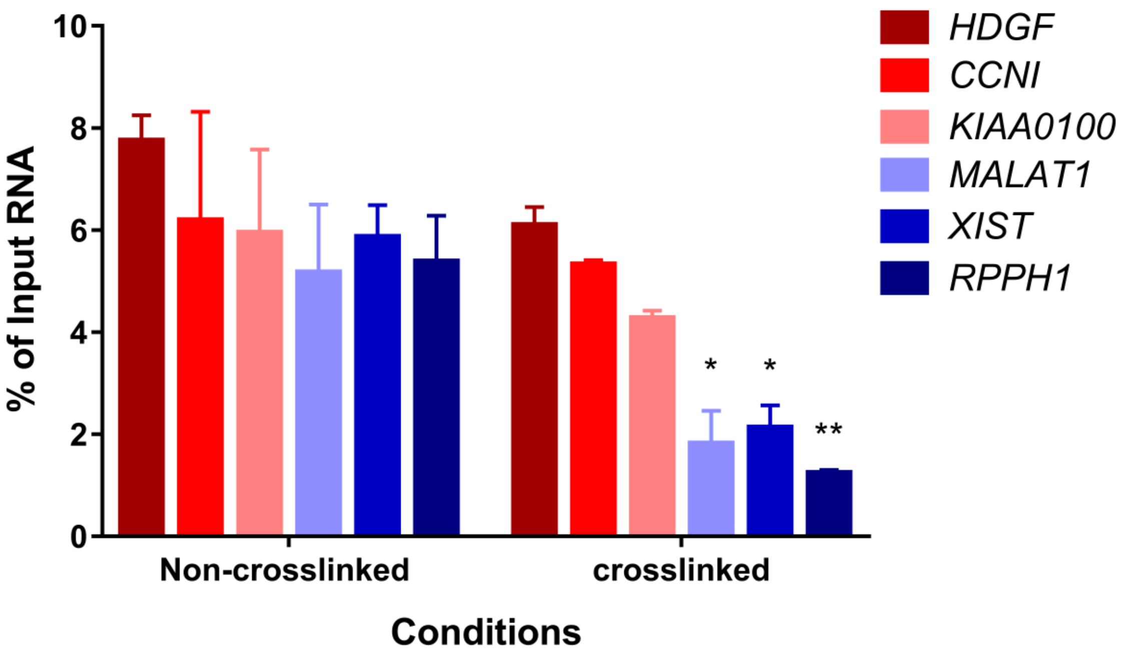

Figure 6. BioTASQ enrichment of G4-RNA using crosslinked verse non-crosslinked samples. RT-qPCR measurements of top ranked genes (HDGF, CCNI, KIAA0100) and lowest-ranked gene (MALAT, XIST, RPPH1) from the G4RP-seq dataset at baseline (with no G4 ligand treatments) condition were tested. Two-way ANOVA was performed against HDGF (top ranked gene) for each gene under each condition. *p<0.05, **p<0.01, ***p<0.001. Experiments were performed with three biological replicates. Error bars represent SEM.

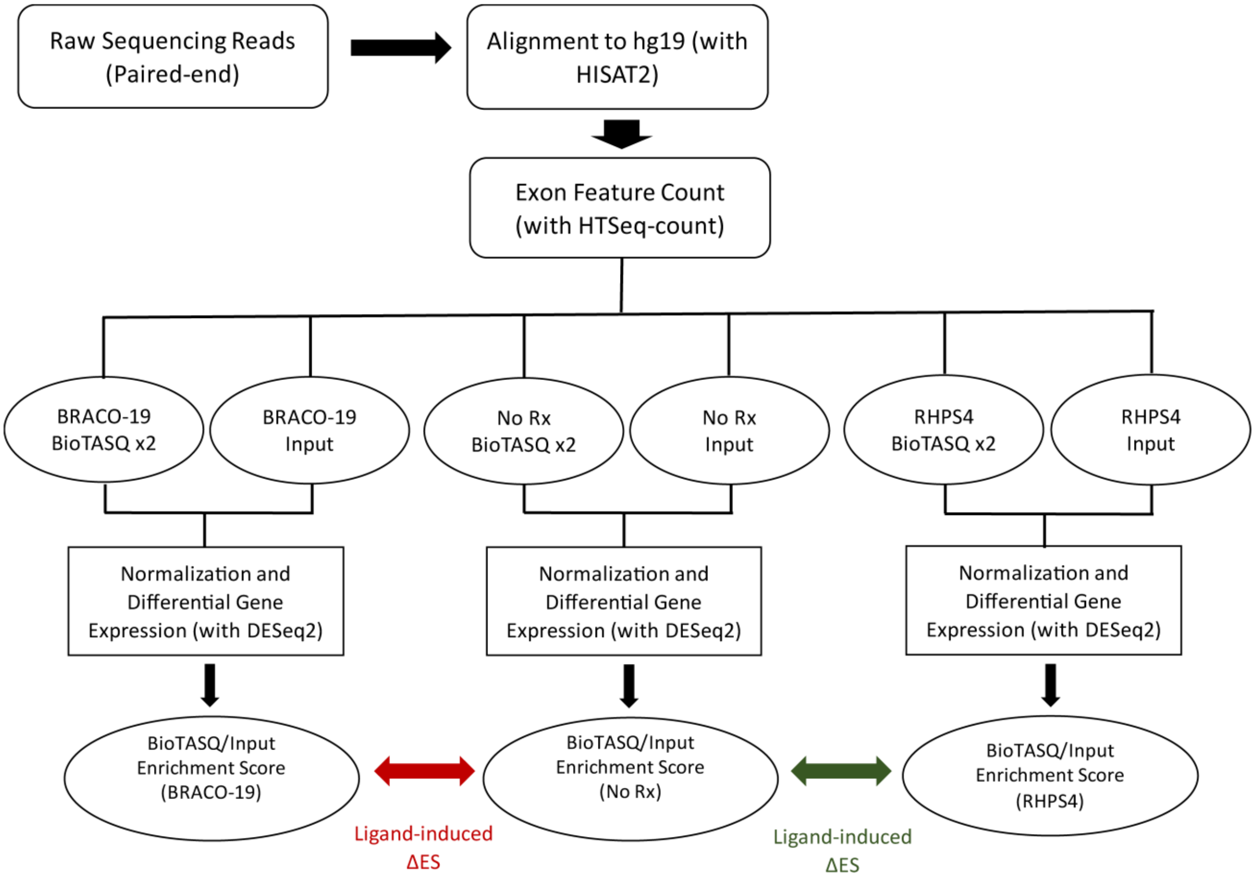

Figure 7. G4RP-seq analysis workflow. Pair-end raw sequencing reads were first aligned to human genome assembly hg19 using HISAT2. The aligned reads within exons were then counted and annotated to genes using HTSeq. The counts were than normalized using DESeq2 before calculating the differential gene expression for each BioTASQ and input pair to obtain the Enrichment Score (ES). BioTASQ/input values (from DESeq2) were compared between conditions to calculate ligand-induced Enrichment Score Change (ΔES) using Excel.

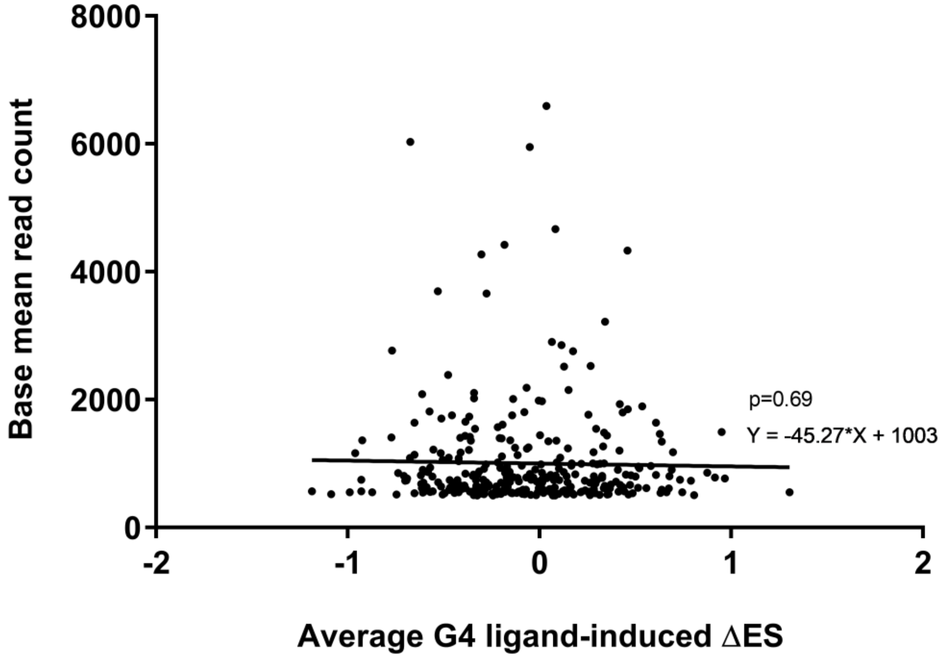

Figure 8. Regression plot of average G4 ligand-induced BioTASQ ΔES versus transcript abundance (as measured by baseline mean read count) (p=0.69, non-significant relationship). Genes with read count of over 8000 were removed to avoid regression being heavily weighted and thus driven by a few points (3 genes out of the 329 removed due to values beyond 35000).

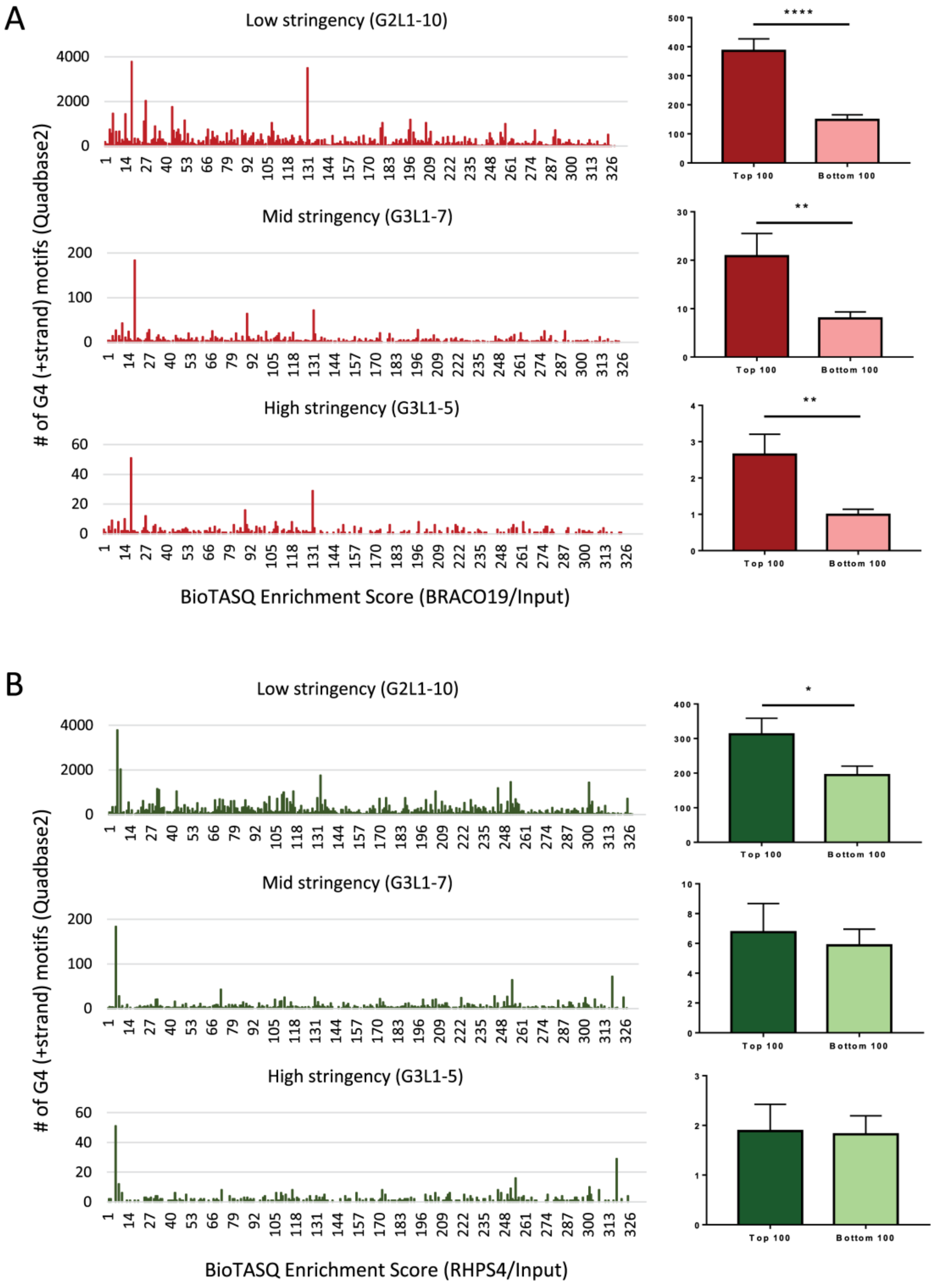

Figure 9. Number of non-overlapping G4 motifs (calculated by Quadbase2) in genes ranked by ES of a) BRACO-19 treated sample (red) and b) RHPS4 treated sample (green). Three different stringency settings are shown: low (G2L1-10), mid (G3L1-7), and high (G3L1-5). Right panel shows the quantification for the top and bottom 100 ranked transcripts. Two-tailed student t-test was performed. *p<0.05, **p<0.01, ***p<0.001. Error bars represent SEM.

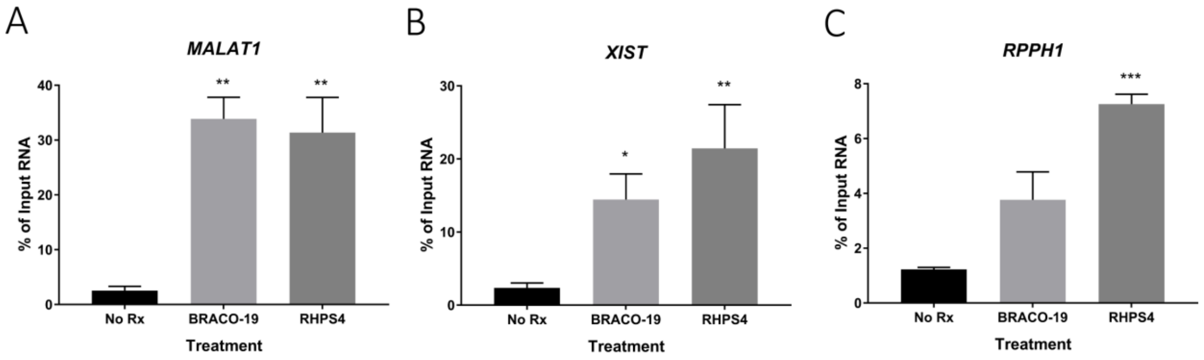

Figure 10. G4RP signal of BioTASQ by RT-qPCR quantification of (A) *MALAT*, (B) *XIST*, (C) *RPPH1* RNA levels in untreated (No Rx), BRACO-19-treated or RHPS4-treated MCF7 cells. Values are normalized to their individual input control used for RIP. Experiments were performed with three biological replicates. One-way ANOVA was performed against non-treated control. *p<0.05, **p<0.01, ***p<0.001. Error bars represent SEM.

-- V. Circular Dichroism results

Circular dichroism (CD) spectra were recorded on a JASCO J-815 spectropolarimeter in a 10mm path-length quartz semi-micro cuvette (Starna). CD spectra were recorded over a range of 220-400nm (bandwidth = 0.5nm, 1nm pitch, 1s response, scan speed = 500nm.mn^{-1}, averaged over 3 scans, zeroed at 340nm) with MALAT1: r[$G_3ATG_3AG_2AG_5TG_3$], XIST: r[$G_2AAG_2AAG_2TTG_2$] and RPPH1: r[$G_2AG_4CCCG_2CG_2$] (10 µM) in 1mL (final volume) of 10mM lithium cacodylate buffer (pH 7.2) + 90mM LiCl/10mM KCl. Thermal difference spectra (TDS) spectra were collected Spectra were recorded on a JASCO V630Bio spectrophotometer and calculated using the same cuvettes (7).

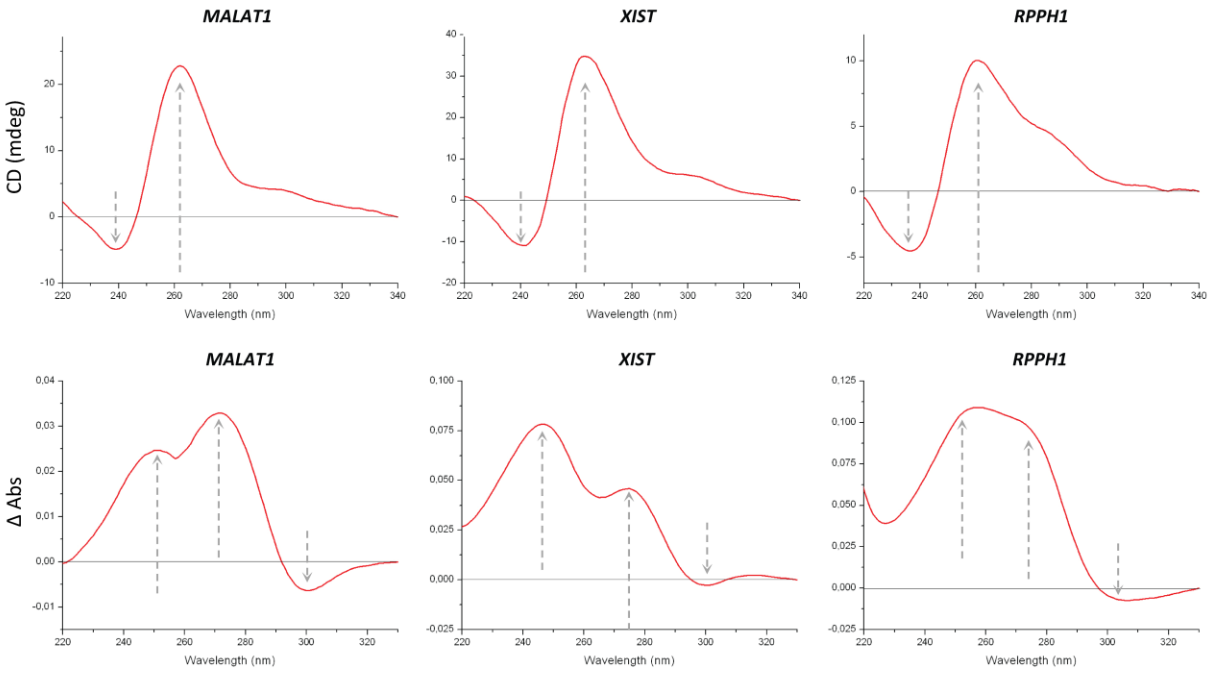

Figure 11. Confirmation of quadruplex topology by circular dichroism (CD) and thermal differential spectra (TDS) investigations of selected quadruplex-forming sequences (QFS) of top ranked gene (MALAT1: r[$G_3ATG_3AG_2AG_5TG_3$] (G-score: 40); XIST: r[$G_2AAG_2AAG_2TTG_2$] (G-score: 21); and RPPH1: r[$G_2AG_4CCCG_2CG_2$] (G-score: 21)). Typical G4-RNA parallel quadruplex signatures were obtained by both CD (positive (260-265nm) and negative peaks (240nm)) and TDS (positive (250-270nm) and negative peaks (295-300nm), dashed arrows).

-- VI. Gene Ontology analysis results

Table 1. Gene Ontology (GO) geneset analysis on baseline G4 levels using the top 100 and bottom 100 highly abundant transcripts ranked by BioTASQ Enrichment Score. The top 5 ranked terms are listed for each category (Cellular component, Biological Process and Molecular Function).

	**Top 100**		**Bottom 100**	
**Rank**	**Cellular Component GO Term**	**Score**	**Cellular Component GO Term**	**Score**
1	cytosolic part	76.46	cytoplasmic stress granule	22.24
2	cytosolic ribosome	69.62	ribonucleoprotein granule	15.79
3	ribosome	55.69	cytoplasmic ribonucleoprotein granule	12.76
4	polysome	50.09	nuclear matrix	12.07
5	cytosolic large ribosomal subunit	49.36	nucleolus	11.3
**Rank**	**Biological Process GO Term**	**Score**	**Biological Process GO Term**	**Score**
1	protein targeting to ER	110.01	ribosomal small subunit export from nucleus	31.5
2	nuclear-transcribed mRNA catabolic process, nonsense-mediated decay	107.93	RNA metabolic process	28.88
3	peptide biosynthetic process	107.5	alternative mRNA splicing, via spliceosome	25.48
4	SRP-dependent cotranslational protein targeting to membrane	100.23	rRNA-containing ribonucleoprotein complex export from nucleus	22.19
5	viral transcription	89.05	ribosomal large subunit export from nucleus	21.62
**Rank**	**Molecular Function GO Term**	**Score**	**Molecular Function GO Term**	**Score**
1	RNA binding	79.86	RNA binding	31.18
2	cadherin binding	43.5	DNA helicase activity	17.57
3	eukaryotic initiation factor 4E binding	31.76	RNA helicase activity	16.9
4	small ribosomal subunit rRNA binding	31.08	protein kinase activator activity	16.79
5	histone methyltransferase activity	28.59	RNA stem-loop binding	15.65

Table 2. Gene Ontology (GO) geneset analysis on filtered gene list (50 mean base count and >1.75 fold change) with BRACO-19- (left) or RHPS4- (right) induced BioTASQ-enrichment. BRACO-19, n=251; RHPS4, n=463. The top 5 ranked terms are listed for each category (Cellular component, Biological Process and Molecular Function).

	**BRACO-19**		**RHPS4**	
**Rank**	**Cellular Component GO Term**	**Score**	**Cellular Component GO Term**	**Score**
1	trans-Golgi network membrane	27.96	nucleolar part	32.64
2	Golgi membrane	19.55	nucleolus	32.41
3	cytoplasmic side of Golgi membrane	19.47	Golgi membrane	22.58
4	extrinsic component of Golgi membrane	19.46	cytoplasmic side of Golgi membrane	22.49
5	Golgi cisterna membrane	19.09	extrinsic component of Golgi membrane	22.46
**Rank**	**Biological Process GO Term**	**Score**	**Biological Process GO Term**	**Score**
1	negative regulation of transcription from RNA polymerase II promoter	40.61	G1/S transition of mitotic cell cycle	33.61
2	negative regulation of mitotic cell cycle	30.78	protein ubiquitination involved in ubiquitin-dependent protein catabolic process	33.59
3	vesicle targeting, rough ER to cis-Golgi	29.64	RNA splicing	24.25
4	endoplasmic reticulum-Golgi intermediate compartment (ERGIC)	29.63	mRNA splice site selection	22.96
5	COPII vesicle uncoating	29.51	RNA export from nucleus	22.44
**Rank**	**Molecular Function GO Term**	**Score**	**Molecular Function GO Term**	**Score**
1	single-stranded RNA binding	25.29	siRNA binding	46.15
2	double-stranded RNA binding	24.74	pre-miRNA binding	46.11
3	misfolded RNA binding	22.11	tRNA binding	45.48
4	RNA binding	22.08	double-stranded RNA binding	44.75
5	base pairing with RNA	22.04	mRNA binding	44.36

-- VII. Sequences used in current study

Table 3. Sequences of oligonucleotides used in the in vitro assays			
FRET-melting Assay	Dual-labeled DNA	F21T	FAM-d[$^{5'}$GGGTTAGGGTTAGGGTTAGGG$^{3'}$]-TAMRA
		F-myc-T	FAM-d[$^{5'}$GAGGGTGGGGAGGGTGGGGAAG$^{3'}$]-TAMRA
		F-kit-T	FAM-d[$^{5'}$CGGGCGGGCGCGAGGGAGGGG$^{3'}$]-TAMRA
		F-DS-T	FAM-d[$^{5'}$TATAGCTATATTTTTTTATAGCTATA$^{3'}$]-TAMRA
	Dual-labeled RNA	F-TERRA-T	FAM-r[$^{5'}$GGGUUAGGGUUAGGGUUAGGG$^{3'}$]-TAMRA
	Unlabeled DNA	ds26	d[$^{5'}$CAATCGGATCGAATTCGATCCGATTG$^{3'}$]
In vitro Pull-down Assay	Labeled DNA	F-myc	FAM-d[$^{5'}$GAGGGTGGGGAGGGTGGGGAAG$^{3'}$]
		F-SRC	FAM-d[$^{5'}$GGGAGGGAGGGCTGGGGG$^{3'}$]
		F-22AG	FAM-d[$^{5'}$AGGGTTAGGGTTAGGGTTAGGG$^{3'}$]
		F-DS	FAM-d[$^{5'}$TATAGCTATATTTTTTTATAGCTATA$^{3'}$]
	Labeled RNA	F-TERRA	FAM-r[$^{5'}$GGGUUAGGGUUAGGGUUAGGG$^{3'}$]
		F-TRF2	FAM-r[$^{5'}$CGGGAGGGCGGGGAGGGC$^{3'}$]
		F-RAS	FAM-r[$^{5'}$GGGAGGGGCGGGUCUGGG$^{3'}$]
	Unlabeled DNA	ds17-1	d[$^{5'}$CCAGTTCGTAGTAACCC$^{3'}$]
		ds17-2	d[$^{5'}$GGGTTACTACGAACTGG$^{3'}$]
		ds26	d[$^{5'}$CAATCGGATCGAATTCGATCCGATTG$^{3'}$]
ESI-MS		Myc	d[$^{5'}$GAGGGTGGGGAGGGTGGGGAAG$^{3'}$]
CD, TDS		MALAT1	r[$G_3ATG_3AG_2AG_5TG_3$]
		XIST	r[$G_2AAG_2AAG_2TTG_2$]
		RPPH1	r[$G_2AG_4CCCG_2CG_2$]

Table 4. Primer set used for RT-qPCR

mRNA	Forward	Reverse	Source
VEGFA	CCTTGCCTTGCTGCTCTACC	AGATGTCCACCAGGGTCTCG	Ref. 8
NRAS	ATGACTGAGTACAAACTGGTGGT	CATGTATTGGTCTCTCATGGCAC	Ref. 8
TERF2	GTACGGGGACTTCAGACAGAT	CGCGACAGACACTGCATAAC	Ref. 8
HPRT1	ACCAGTCAACAGGGGACATAA	CTTCGTGGGGTCCTTTTCACC	Ref. 8
MALAT1	AAAGCAAGGTCTCCCCACAAG	GGTCTGTGCTAGATCAAAAGGCA	Ref. 9
XIST	GGTCTGTGCTAGATCAAAAGGCA	AGCTCCTCGGACAGCTGTAA	Ref. 10
RPPH1	GAGCTGAGTGCGTCCTGTC	TCAGGGAGAGCCCTGTTAGG	Ref. 11
HDGF	AACAACCCTACTGTCAAGGCT	TCTTCAACGCTCCTTTCTCGT	Ref. 8
CCNI	AAGTGAATGTGCGGAAAATGC	GGCCAGCCATTGAATTACTTCAT	Ref. 8
KIAA0100	GGTGGGCATTAGCAGTCGG	ATACCACGCTTGTGTTCTCCA	Ref. 8

Supplementary Reference

1. Haudecoeur, R., Stefan, L., Denat, F. & Monchaud, D. A Model of Smart G-Quadruplex Ligand. *J. Am. Chem. Soc.* **135,** 550-553 (2013).
2. Laguerre, A., Levillain, M., Stefan, L., Haudecoeur, R., Katranji, F., Pirrotta, M., & Monchaud, D. Synthetic G-Quartets as Versatile Nanotools for the Luminescent Detection of G-Quadruplexes. *CHIMIA International Journal for Chemistry*, **69**, 530-536 (2015).
3. Rousselin, Y., Sok, N., Boschetti, F., Guilard, R., & Denat, F. Efficient Synthesis of New C-Functionalized Macrocyclic Polyamines. *Eur. J. Org. Chem.* **2010**, 1688-1693 (2010).
4. De Cian, A., Guittat, L., Kaiser, M., Saccà, B., Amrane, S., Bourdoncle, A., Alberti, P., Teulade-Fichou, M.P. & Mergny, J. L. Fluorescence-based melting assays for studying quadruplex ligands. *Methods*, **42**, 183-195 (2007).
5. Rosu, F., Gabelica, V., Houssier, C., & De Pauw, E. Determination of affinity, stoichiometry and sequence selectivity of minor groove binder complexes with double-stranded oligodeoxynucleotides by electrospray ionization mass spectrometry. *Nucleic Acids Res.*, **30**, e82-e82 (2002).
6. Yang, S.Y., Amor, S., Laguerre, A., Wong, J.M. & Monchaud, D. Real-time and quantitative fluorescent live-cell imaging with quadruplex-specific red-edge probe (G4-REP). *Biochim. Biophys. Acta*. **1861,** 1312-1320 (2017).
7. Mergny, J.L. et al. Thermal difference spectra: a specific signnature for nucleic acid structures. *Nucleic Acids Res*. **33**, e138-e138 (2005).
8. Wang, X., Spandidos, A., Wang, H. & Seed, B. PrimerBank: a PCR primer database for quantitative gene expression analysis, 2012 update. *Nucleic Acids Res.* **40,** D1144-D1149 (2011).
9. Ma, K.X. et al. Long noncoding RNA MALAT1 associates with the malignant status and poor prognosis in glioma. *Tumor Biol.* **36,** 3355-3359 (2015).
10. Gilbert, S.L., Pehrson, J.R. & Sharp, P.A. XIST RNA associates with specific regions of the inactive X chromatin. *J. Biol. Chem*. **275,** 36491-36494 (2000).
11. Cai, Y. et al. Rpph1 upregulates CDC42 expression and promotes hippocampal neuron dendritic spine formation by competing with miR-330-5p. *Front. Mol. Neurosci.* **10,** 27 (2017).
