# Supplementary material for: Transcriptome-wide identification of transient RNA G-quadruplexes in human cells
Source: Nat Commun. 2018 Nov 9;9:4730. doi: 10.1038/s41467-018-07224-8 (PMC6226477; doi:10.1038/s41467-018-07224-8)
Supplement: Supplementary file 3 — Description of Additional Supplementary Items [file 41467_2018_7224_MOESM3_ESM.docx]

**Description of Additional Supplementary File**

File Name: Supplementary Data 1

File Description: A) Full initial list of gene transcripts (>50 base mean count) with corresponding BioTASQ Enrichment Score (ES) shown in log2(fold change) value. Enrichment Score Change (ΔES) is shown for BRACO-19 (BRACO-19/NoRx) and RHPS4 (RHPS4/NoRx). Average G4 ligand-induced changes are calculated from the average of the ΔES from two treatments. The HUGO information is listed, as well as Gene %GC content. B) Filtered list of gene transcripts based on BRACO19-induced ΔES (>1.75). C) Filtered list of gene transcripts based on RHPS4-induced ΔES (>1.75). D) Filtered list of gene transcripts by base mean count >500. This list also includes Quadbase2 pG4 motif density calculation for each gene with mid stringency (G3L1-7), low stringency (G2L1-12) and high stringency (G3L1-3).
